# Robustness of spiking Deep Belief Networks to noise and reduced bit precision of neuro-inspired hardware platforms

**DOI:** 10.3389/fnins.2015.00222

**Published:** 2015-07-09

**Authors:** Evangelos Stromatias, Daniel Neil, Michael Pfeiffer, Francesco Galluppi, Steve B. Furber, Shih-Chii Liu

**Affiliations:** ^1^Advanced Processor Technologies Group, School of Computer Science, University of ManchesterManchester, UK; ^2^Institute of Neuroinformatics, University of Zurich and ETH ZurichZurich, Switzerland; ^3^Centre National de la Recherche Scientifique UMR 7210, Equipe de Vision et Calcul Naturel, Vision Institute, UMR S968 Inserm, CHNO des Quinze-Vingts, Université Pierre et Marie CurieParis, France

**Keywords:** Deep Belief Networks, spiking neural networks, SpiNNaker, noise robustness, neuro-inspired hardware

## Abstract

Increasingly large deep learning architectures, such as Deep Belief Networks (DBNs) are the focus of current machine learning research and achieve state-of-the-art results in different domains. However, both training and execution of large-scale Deep Networks require vast computing resources, leading to high power requirements and communication overheads. The on-going work on design and construction of spike-based hardware platforms offers an alternative for running deep neural networks with significantly lower power consumption, but has to overcome hardware limitations in terms of noise and limited weight precision, as well as noise inherent in the sensor signal. This article investigates how such hardware constraints impact the performance of spiking neural network implementations of DBNs. In particular, the influence of limited bit precision during execution and training, and the impact of silicon mismatch in the synaptic weight parameters of custom hybrid VLSI implementations is studied. Furthermore, the network performance of spiking DBNs is characterized with regard to noise in the spiking input signal. Our results demonstrate that spiking DBNs can tolerate very low levels of hardware bit precision down to almost two bits, and show that their performance can be improved by at least 30% through an adapted training mechanism that takes the bit precision of the target platform into account. Spiking DBNs thus present an important use-case for large-scale hybrid analog-digital or digital neuromorphic platforms such as SpiNNaker, which can execute large but precision-constrained deep networks in real time.

## 1. Introduction

Deep neural networks represent the state-of-the-art solution for virtually all relevant machine learning, computer vision, and speech recognition benchmarks (Schmidhuber, 2015). Their advantage over shallow architectures lies in their ability to extract hierarchies of increasingly abstract relevant features, which give rise to a data representation that lends itself for task-specific optimization. Whereas convolutional networks (LeCun et al., 1998; Sermanet et al., 2014) currently outperform other architectures on many vision tasks, the alternative architecture of Deep Belief Networks (DBNs) (Hinton and Salakhutdinov, 2006) remains very popular due to its ability to learn from large unlabeled datasets (Le et al., 2012), and because of its dual role as classifier and generative model of the data. In addition, DBNs have been shown to improve theoretical performance bounds by adding additional layers of neurons (Hinton and Salakhutdinov, 2006). Although training larger and larger networks is currently the focus of academic and industrial research, this has led to growing demands on hardware platforms for deep learning. While training remains the biggest bottleneck, and some of the biggest networks trained to date have required days or weeks on high-performance computing infrastructure (Devin and Mao, 2012; Le et al., 2012), the sheer size of the resulting network calls for special purpose or GPU hardware acceleration to make the system run close to real-time (Farabet et al., 2011). However, low latency and real-time execution are key demands for mobile and robotic systems, which have limited computing resources and power but require quick system responses.

A recently proposed solution to overcome the energy demands, communication overhead, and high response latencies of DBNs is to transform them into spiking neural networks, thereby exploiting the energy efficiency of event-based updates and communication (O'Connor et al., 2013). Furthermore, the proposed framework, which has shown the desired low latency and high efficiency is targeted for implementation on event-based neuromorphic hardware platforms. Event-driven networks can have higher energy efficiency because a clock is not used in the network simulation, and not every neuron updates in every time step. The efficiency of the event-driven platform TrueNorth (Merolla et al., 2014b) is around 46 GSops/W, where Sops stands for *synaptic operations per second*.

A first hardware implementation of a spiking DBN was achieved on an event-driven Field-Programmable Gate Array (FPGA), yielding an implementation of spiking DBNs called Minitaur (Neil and Liu, 2014). The energy efficiency of Minitaur measured on the digit classification task using the MNIST database is around 12.48–18.73 MSops/W while the CPU efficiency for a Core 2 Duo running the same DBN network is 1.68 MSops/W. Based on Minitaur, we have recently presented an even more efficient implementation of deep neural networks on SpiNNaker (Stromatias et al., 2015a,b), a hardware platform optimized for scalable event-based simulations (Furber et al., 2014). This platform has a biologically-inspired architecture designed to enable low-power and low-latency massively parallel large-scale simulations of heterogeneous models of spiking neurons in real-time. A single SpiNNaker board with 48 Application-Specific Integrated Circuit chips (SpiNN-5), which is the building block for creating larger SpiNNaker machines, delivers up to 54.27 MSops/W in real-time (Stromatias et al., 2013). Because of this massive parallelism and SpiNNaker's optimized communication infrastructure, spiking network implementations on SpiNNaker have beneficial scaling properties, allowing to overcome the latency, communication, and energy issues of conventional computing systems for real-time applications.

However, gains in energy efficiency should not be outweighed by losses in classification performance due to computation with spikes instead of real numbers, or due to limitations of the hardware compared to conventional computers. The hope is that in networks of such large size, numerical imprecision would rather cancel out than accumulate. In the conversion of O'Connor et al. (2013) from digital to spiking DBNs in software only small performance losses and overall very good performance was reached. These results also hold for Minitaur (Neil and Liu, 2014). In the present article we present a full characterization of software implementations of spiking DBNs, developing a set of case studies to determine the impact of the hardware bit precision, the input noise, weight variance, and combinations on the classification performance of a deep network for handwritten digit recognition. These studies provide important guidelines for informing current and future efforts to develop custom large-scale digital and mixed-signal spiking network platforms such as SpiNNaker, TrueNorth, Neurogrid, Bluehive, and BrainScales (Moore et al., 2012; Pfeil et al., 2012, 2013; Benjamin et al., 2014; Merolla et al., 2014a,b). Although the present study addresses only the scenario of off-chip learning, it also provides guidelines for the design of hardware learning circuits that can train synaptic weights in DBNs (Mitra et al., 2009; Neftci et al., 2014). In particular, our results show that the performance of DBNs on low-precision platforms can be dramatically improved by an adapted training scheme that directly targets learning of low bit precision representations, rather than rounding the weights obtained from full-precision software simulations. Hence, our results suggest that spiking DBNs can be realized on limited precision hardware platforms without drastic performance loss, and thus offer an excellent compromise between accuracy and low-power, low-latency execution.

The investigation of the impact of weight variance and precision on performance is particularly relevant for future hybrid analog-digital spiking network implementations and for new memristive technologies that are being proposed to implement synaptic states and corresponding spike-timing based learning rules (Alibart et al., 2012; Kuzum et al., 2012; Cruz-Albrecht et al., 2013; Serrano-Gotarredona et al., 2013). Our results are in good agreement with a recent study of the impact of weight precision for bistable memristive synapses in a probabilistic learning task, which has revealed a surprisingly low level of precision required for reliable learning of handwritten digit patterns (Bill and Legenstein, 2014).

This paper is structured as follows: Section 2 describes the theory of spike-based DBNs, training methods, and methods to reduce bit precision and introduce parameter noise. Section 3 presents investigations on the impact of bit precision of the hardware platform and input noise on the classification performance of a spike-based DBN using the MNIST digit database. These investigations include the impact of silicon mismatch on the coding of the weight parameters, and present novel off-line training methods to overcome reduced hardware precision. Lastly, Section 4 concludes with discussions regarding the results of this study and the impact on future hardware implementations.

## 2. Materials and methods

### 2.1. Spiking deep belief networks

**Figure 1:**
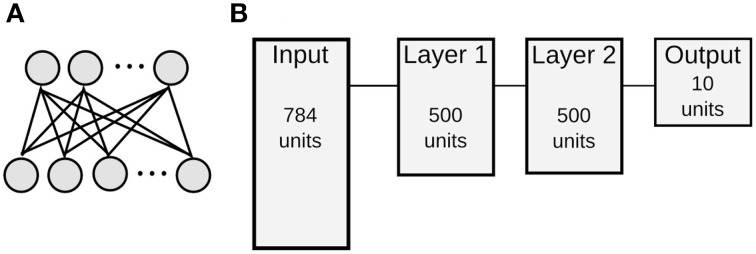
**Architecture of RBMs and DBNs**. **(A)** Architecture of a single Restricted Boltzmann Machine with full connectivity between visible units (bottom) and hidden units (top), but no connections within the same layer. **(B)** Topology of a DBN for MNIST classification consisting of one input layer with 784 neurons, two hidden layers with 500 neurons each, and a 10 neuron output layer. This is abbreviated as 784-500-500-10 architecture.

We use the formalism for training and executing spiking DBNs developed by O'Connor et al. (2013). DBNs are multi-layered neural networks, in which each layer pair is formed by a Restricted Boltzmann Machine (RBM). The two layers of visible and hidden units of a RBM are fully and recurrently connected, but there are no connections between neurons of the same layer (Figure 1). In a conventional RBM, each unit is a stochastic binary neuron, and the probability to turn on is given by a sigmoid function applied to the weighted sum of its inputs. Layers are trained one after another with an unsupervised rule called Contrastive Divergence (CD) (Hinton and Salakhutdinov, 2006). When training of one layer is finished, the output of the hidden units of one layer serves as the input to visible units of the subsequent layer. Supervised learning is used at the top level, where a label is jointly trained with the input, and this serves as the output of the network.

Spiking DBNs use a training procedure that is very similar to conventional DBN training, discussed in Section 2.2, to yield suitable connection weights for a network of spiking neurons to solve the intended task. Once these weights $\vec{w}$ of each RBM have been fixed, the Leaky Integrate-and-Fire (LIF) neurons follow the standard dynamics for the membrane potential *V*, described as

(1) $$\tau_{m}\frac{dV}{dt}=E_{L}-V+R_{m}I,$$

where τ_*m*_ is the membrane time constant, *E*_*L*_ the resting potential, and *R*_*m*_ the membrane resistance. The input current *I* is computed as

(2) $$I=\sum_{i\;=\;1}^{n}w_{i}\sum_{j\;=\;1}^{m_{i}}\delta (t-t_{ij}),$$

where *n* is the number of incoming synapses, *w*_*i*_ is the weight of synapse *i*, *m*_*i*_ is the number of spikes arriving at that synapse, and δ(*t*) is a Dirac delta function which is zero except for the firing times *t*_*ij*_ of the *i*^th^ input neuron. Once the membrane potential *V* crosses the threshold voltage *V*_thresh_ a spike is generated, the membrane potential is reset to *V*_reset_ and the neuron is not allowed to fire during the refractory period *T*_ref_. Default values of the parameters used in simulations are defined in Table 1.

**Table 1:** **Default parameters of the Leaky Integrate-and-Fire Model used in simulations**.

**Parameters**	**Values**	**Units**
τ_*m*_	5.0	s
*T*_ref_	2.0	ms
*V*_reset_	0.0	mV
*V*_thresh_	1.0	mV

### 2.2. Training spiking deep belief networks

Training of DBNs targeting a spiking network implementation is described in detail in O'Connor et al. (2013). The following section provides a brief summary of the most important differences to conventional CD training. The key idea is to use an accurate rate-based approximation of the firing rates of LIF neurons, and translate this into activation probabilities, which can be used in the CD updates. We use the so-called Siegert approximation (Jug et al., 2012), which approximates the output firing rate of a LIF neuron receiving both inhibitory and excitatory inputs. Let $\vec{\rho }$_*i*_ and $\vec{\rho }$_*e*_ be the vectors of inhibitory and excitatory input rates, and ($\vec{w}$_*i*_, $\vec{w}$_*e*_) be the corresponding weights. In order to compute the expected output rate of the LIF neuron, a number of auxiliary variables first needs to be computed. For completeness, we provide the full equations, but refer to previous work for the derivation and interpretation of each variable (Siegert, 1951; Jug et al., 2012):

$$\begin{array}{l} \mu_{Q}=\tau \sum^{\text{​}}({\vec{w}}_{e}{\vec{\rho }}_{e}+{\vec{w}}_{i}{\vec{\rho }}_{i})\;\sigma_{Q}^{2}=\frac{\tau }{2}\sum^{\text{​}}({\vec{w}}_{e}^{2}{\vec{\rho }}_{e}+{\vec{w}}_{i}^{2}{\vec{\rho }}_{i})\\ \;\;\;\;\;\;\;\;\;\;\;\;\;\;\;\;\;\;ϒ=V_{reset}+\mu_{Q}\;\;\Gamma =\sigma_{Q}\\ \;\;\;\;\;\;\;\;\;\;\;\;\;\;\;\;\;\;\;\;\;\;k=\sqrt{\tau_{syn}/\tau }\;\gamma =|\zeta (1/2)| \end{array}$$

Here, τ_syn_ denotes the synaptic time constant (for our purposes considered to be zero), and ζ is the Riemann zeta function. Then the average firing rate ρ_out_ of the neuron with reset potential *V*_reset_, threshold voltage *V*_thresh_, and refractory period *T*_ref_ can be computed as (Jug et al., 2012)

(3) $$\begin{array}{l} \rho_{\text{out}}=(T_{\text{ref}}+\frac{\tau }{\Gamma }\sqrt{\frac{\pi }{2}}\;\cdot \;\int_{V_{\text{reset}}+k\gamma \Gamma }^{V_{\text{thresh}}+k\gamma \Gamma }\exp\left[\frac{{\left(u-ϒ\right)}^{2}}{2\Gamma^{2}}\right]\cdot \\ \;{\cdot \;\left[1+\text{erf}\left(\frac{u-ϒ}{\Gamma \sqrt{2}}\right)\right]du)}^{-1}. \end{array}$$

Using this approximation of firing rates allows a direct translation between the analog activation probabilities required for CD training and the resulting firing rates of a spiking neuron with those weights. During training of the spiking DBN, the Siegert approximation is used as the nonlinearity of the neuron instead of a sigmoidal function. The predicted rate ρ_out_ in Equation (3) can be converted into a probability by normalizing with the maximum firing rate 1/T_ref_. This allows sampling the activation probabilities, as is done in standard contrastive divergence learning with with continuous-valued units. Specifically, the weight update in contrastive divergence for spiking networks computes the data- and model-driven activities of the visible and hidden layer using the Siegert approximation, and then computes the weight update as usual in RBM training. Let *V*_data_ be the activity of the visible units driven by the input data (or activity of the hidden layer below). Then the data-driven activity of the hidden layer, given the full weight matrix *W* connecting the visible and hidden layer, is

$$H_{\text{data}}=\rho_{\text{out}}(V_{\text{data}},W)\;\cdot \;T_{\text{ref}}$$

The model-driven activity of the visible and hidden layers, obtained via Gibbs sampling, is then given as

$$\begin{array}{l} V_{\text{model}}=\rho_{\text{out}}(H_{\text{data}},W^{T})\;\cdot \;T_{\text{ref}},\\ H_{\text{model}}=\rho_{\text{out}}(V_{\text{model}},W^{T})\;\cdot \;T_{\text{ref}} \end{array}$$

and the weight update Δ*w* is

(4) $$\Delta w(W)=\alpha \;\cdot \;(H_{\text{data}}^{T}V_{\text{data}}-H_{\text{model}}^{T}V_{\text{model}}),$$

where α is the learning rate. We parameterize Δ*w* by the weight matrix *W*, because later different weight matrices with different bit precisions will be used to calculate the activities of hidden and visible layers. After training, the parameters and weights are kept unchanged, but instead of sampling every time step, the units generate Poisson spike trains with rates computed by the Siegert formula (Equation 3). In O'Connor et al. (2013) we have shown that this results in equivalent spiking implementations of RBMs and DBNs, which perform similarly to conventional networks with the same architecture.

### 2.3. Database, image conversion to spikes, and input noise generation

**Figure 2:**
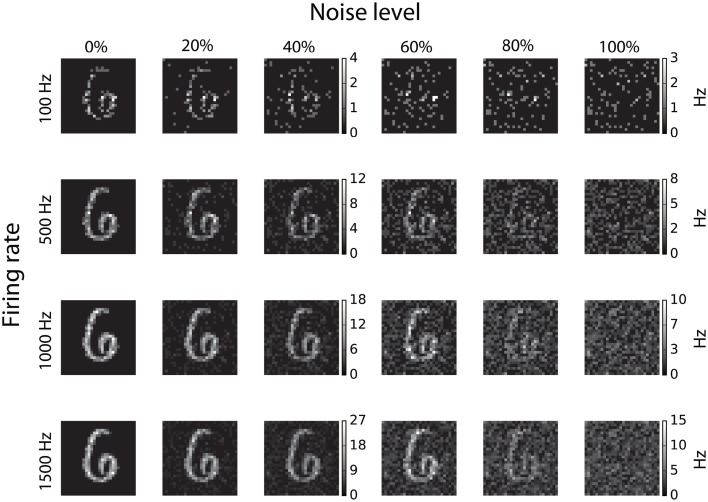
**Conversion of static images to spike-trains and introduction of noise**. Each row represents different input rates ranging from 100 to 1500 Hz, while the columns show different percentages of input noise, from 0 up to 100%.

Training and testing of spiking DBNs was carried out on the well-known MNIST database of handwritten digits (LeCun et al., 1998), which consists of 70,000 28 × 28 gray-scale pixel images, of which 10,000 are used as a test set. In order to convert the static images to spike trains, each pixel of an MNIST image is converted to a Poisson spike-train with a rate proportional to its intensity, while all firing rates are scaled such that the total firing rate of the population is constant (O'Connor et al., 2013). In order to determine the impact of input noise on the performance of DBNs, noise is introduced into the spike-train representation of each image by redistributing a percentage of spikes randomly across the whole input population (Neil and Liu, 2014). The resulting digits with different noise levels are shown in Figure 2, where each column represents different levels of noise starting from 0% redistribution in the first column, to 100% in the last column.

### 2.4. Conversion from double to lower precision representations

In order to simulate spiking DBNs implemented on digital architectures with limited hardware resources (Moore et al., 2012; Furber et al., 2014; Merolla et al., 2014b; Neil and Liu, 2014), the weights learned with double floating-point precision during training are converted to lower bit-precision representations.

Throughout this paper we use the notation Q*m.f* to indicate a fixed-point format where *m* is the number of bits in the integer part, including the sign bit, followed by a notional binary point, and *f* is the number of bits in the fractional part. This format is a bit-level format for storing a numeric value.

Contrary to the fixed-point format, the double-precision floating-point numbers according to the IEEE 754 standard have a 64-bit word length of which 52 bits are used to store for the fraction, 11 bits for the exponent and one bit for the sign. Moreover a floating-point unit (FPU) is needed for computations with floating-point numbers, which results in increased area of the hardware design and higher energy costs.

The bit precision of the synaptic weights was set by keeping the number of the integer bits constant to a value that is capable of holding the maximum and minimum weight value of a particular DBN, while the number of bits in the fractional part is varied from eight bits down to one bit. The double precision floating-point weights *W*_*H*_ are converted to lower-precision representation *W*_*L*_ using the conversion

(5) $$W_{L}=\text{round}(2^{f}\;\cdot \;W_{H})\;\cdot \;2^{-f}$$

where *W_*H*_* are the original double floating-point weight values of the trained DBN, and 2^−*f*^ is the resolution of the lower precision representation.

### 2.5. Introducing weight variability

In order to investigate the effect of mismatch when mapping the spiking DBN to mixed-mode analog/digital multi-neuron transistor circuits, we considered a particular synaptic circuit known as the digital-to-analog converter (DAC) synapse (Serrano-Gotarredona et al., 2008; Wang and Liu, 2013). In this circuit, the synaptic weight is represented as a current. This synapse has a maximum current and the number of bits in the DAC sets the resolution of the synaptic current (weight). In considering the effect of mismatch due to silicon fabrication, we assume that the maximum current of each DAC synapse is sampled from a Gaussian distribution. The variability is controlled by defining the coefficient of variation (CV) of the distribution from which weights are sampled, ranging from 10 to 40%. In this analysis, we do not include calibration using the DAC to account for the mismatch in the maximum current. Calibrating the DAC synapse to reduce the transistor mismatch even over the network of the size used in this work would require a long testing time.

### 2.6. Event-based platforms

Spiking DBNs were recently implemented on portable digital hardware platforms and neurally inspired hardware, which dissipate much less power than conventional processors. In particular, this has been shown by an FPGA implementation, dubbed *Minitaur* (Neil and Liu, 2014), and on the SpiNNaker spiking neural platform (Stromatias et al., 2015a,b). The software simulation results in this work are validated by comparing to spiking DBNs running on the SpiNNaker platform.

The SpiNNaker platform (Furber and Temple, 2008; Furber et al., 2014) is a biologically inspired Application Specific Integrated Circuit (ASIC) designed to enable real-time simulations of heterogeneous models of spiking neurons and synapses. The fundamental component of the system is the SpiNNaker Chip. It contains 18 identical and fully programmable ARM9 cores, each of which has access to a local 96KBs tightly-coupled memory (TCM), executing the runtime neural kernels and storing the neural parameters. All the cores on a chip have access to a shared 128 MBs off-die SDRAM memory, where all relevant synaptic information is stored and retrieved upon the arrival of an event. The SpiNNaker platform has no hardware floating-point support, thus the neuron states and synapses are computed using fixed-point arithmetic (Furber et al., 2013, 2014). This was part of the design specification in order to further improve the energy efficiency of the platform. The key innovation of the machine is in its bespoken communication infrastructure inspired by spike communication. The design allows delivery of large volumes of very small packets to be communicated across the machine, which makes it different from conventional super-computers which tend to transfer data in large lumps.

The building block of SpiNNaker machines is a single PCB comprising 48 SpiNNaker chips. This has proven to be capable of simulating a quarter million neurons with more than 80 million synapses. In terms of activity, the tested system is able to generate a dynamic activity of 1.76 billion synaptic events (SE) per second while dissipating <1 W per chip (<40 W for the whole board, Stromatias et al., 2013). Interconnecting more of these boards together will form the final SpiNNaker machine, which will utilize ≈60,0000 chips, for a total of more than a million ARM cores, and it aims at simulating 1% of the human brain in real-time (Furber et al., 2013).

The networks simulated using the Brian software package can easily be mapped on to the SpiNNaker platform through an interface with a high-level simulator independent neural specification language called PyNN (Davidson et al., 2009) by utilizing a tool named Partitioning And Configuration MANagement (PACMAN) (Galluppi et al., 2012).

## 3. Results

The performance of spiking DBNs with reduced precision or weight variability is assessed on a common benchmark task, the classification of handwritten digits from the MNIST dataset (LeCun et al., 1998). The MNIST dataset is divided into a 60,000 digit training set, and a 10,000 digit test set. The conversion of the 28 × 28 gray-scale pixel images into spike trains and training of DBNs are described in Section 2.3. Simulation results of the spike-based DBN were obtained using the Brian spiking neural network simulator (Goodman and Brette, 2008).

In the following we characterize the impact of reduced bit precision and noise on the classification performance of spiking DBNs. In Section 3.1 the impact of lower precision or higher input noise levels on fully trained networks during testing is demonstrated, along with an investigation of the possible reasons for the resulting performance curves in Section 3.2. Section 3.3 shows how variance on the weight parameters due to transistor mismatch affects the network performance for a particular analog synaptic circuit. The results in Section 3.1 are verified by a comparison between the performance of a software simulation in Brian vs. that of the corresponding implementation on the digital hardware SpiNNaker platform (Section 3.4). Finally, Section 3.5 presents a new method to avoid performance loss on lower-precision platforms by introducing a training algorithm that takes into account the lower bit precision of the hardware and produces weights which increase the overall performance of the network using the lower bit precision weights.

### 3.1. Robustness to reduced bit precision of fixed-point synapses

**Figure 3:**
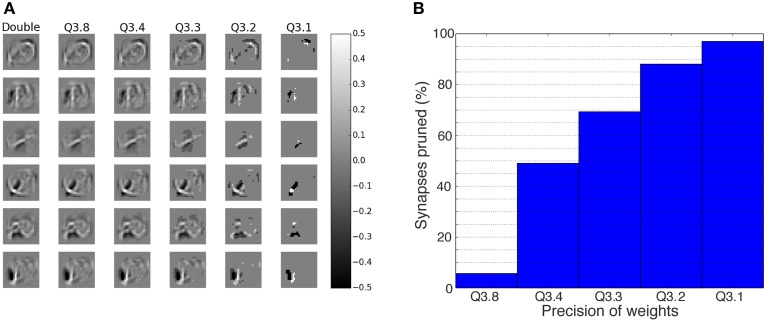
**Impact of weight bit precision on the representations within a DBN**. **(A)** The receptive fields of the first six neurons (rows) in the first hidden layer. The first column shows the weights for double precision, while the remaining columns show the weights for different fixed-point schemes, using the format Q*m*.*f*, where *m* is the number of bits representing the integer part and *f* the number of bits representing the fractional part. The effect of truncation of the synapses is visible to the eye for the case of *f* = 4 bits and less. **(B)** Percentage of synapses from all layers that are set to zero due to the reduction in bit precision for the fractional part. For lower precisions, the majority of the synapses is set to zero and thus becomes obsolete.

Reduction in bit precision will reduce the resources needed on a digital chip (Underwood, 2004). If the performance of the network is maintained even when the bit precision drops, then a larger network can be implemented for the same amount of resources. The impact of the bit precision on the trained double precision floating-point weights can be seen in Figure 3A. Shown in the figure are the receptive fields of six of the neurons in the first hidden layer (Layer 1) for different fixed-point precisions of the synapses, ranging from double precision in the first column, to weights down to one bit for the fractional part in the last column. The figure shows that a lot of the structure in the receptive fields is still retained even with a bit precision of down to *f* = 4 bits. Figure 3B shows the percentage of synapses that were set to zero due to the bit reduction in the fractional part. Most compelling is that even at Q3.4, almost 50% of the weights are zero, which means these synapses are obsolete and can be pruned, thereby reducing the memory resources even further.

**Figure 4:**
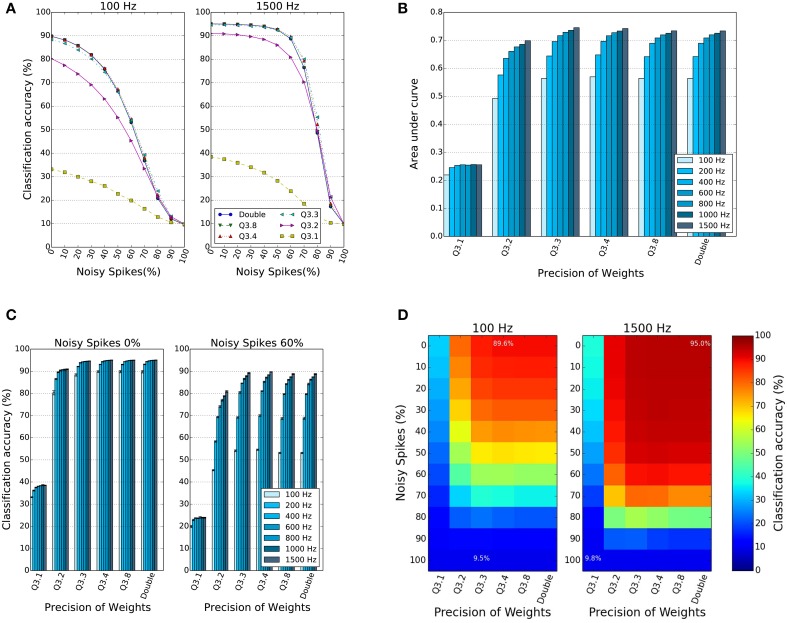
**Effect of reduced weight bit precision and input noise on the classification accuracy (CA)**. **(A)** CA as a function of input noise and bit precision of synaptic weights for two specific input spike rates of 100 and 1500 Hz. Results over four trials. **(B)** Normalized area under curve in **(A)** for different percentages of input noise, input firing rates and weight bit precision. Higher values mean higher accuracy and better robustness to noise. **(C)** CA as a function of the weight bit resolution for different input firing rates and for two different noise levels, 0 and 60%. **(D)** CA as a 2D function of the bit resolution of the weights and the percentage of input noise for 100 and 1500 Hz input rate. The results confirm that spiking DBNs with low precision weights down to *f* = 3 bits can still reach high performance levels and tolerate high levels of input noise.

Figure 4A shows the classification accuracy (CA) of the network for different percentages of noise in the input spikes and over different input rates. The different curves within the two sets of plots in Figure 4A show the CA as a function of the percentage of input noise spikes. The two panels show results for two different input rates, 100 and 1500 Hz, which represent the total number of input spikes summed over all input neurons in the stimulus duration of 1 s. For both firing rates, the CA curves drop as the percentage of input noise spikes increases, but for 1500 Hz input the performance stays almost constant until input noise levels of 50% are reached. The different curves show the behavior for different bit precisions of the weights. The peak performance (without noise), as well as the CA for higher input noise levels stays remarkably constant for bit precisions as low as *f* = 3. In general, reduced precision does affect the CA performance, but the peak CA value obtained for double bit precision weights decreases only by around 5% (from 95 to 90%), even when the bit precision drops to *f* = 2. In order to summarize the noise robustness for different precisions and firing rates, the area under the curve in Figure 4A is computed, since larger area indicates both high peak performance and a slow drop-off in performance. Figure 4B shows the area under the curves in Figure 4A as a function of the input firing rate and across five different bit precision values. The results show similar trends for different bit precision levels, and a similar increase in performance and noise tolerance for higher input firing rates. This is also illustrated in Figure 4C, where it can be seen how the CA for different bit precisions changes as the input rates are increased from 100 up to 1500 Hz, and for two different input noise levels. A drop-off in CA of around 5% to 10% for the same input rate can be observed for 60% noise spikes. The 2D plots in Figure 4D finally illustrate that there is a large range of input noise levels and bit precisions at which high performance for two different input rates can be reached. In particular, the results show that surprisingly the performance and noise robustness curves are almost identical for bit precisions down to *f* = 3 bits in all subplots. Even a synaptic weight in Q3.2 representation, which requires <10% of the memory resources for double precision weights gives a reasonable peak CA of 91% for low noise levels. In all subplots (Figures 4A–D), only the Q3.1 representation shows a dramatic drop in performance.

### 3.2. Distribution of reduced bit precision weights

In order to understand better the surprising tolerance of spiking DBNs to reduced weight precision and high input noise levels, the impact of the reduction of precision on the distribution of weights, firing rates, and neuron activations in the network is investigated.

**Figure 5:**
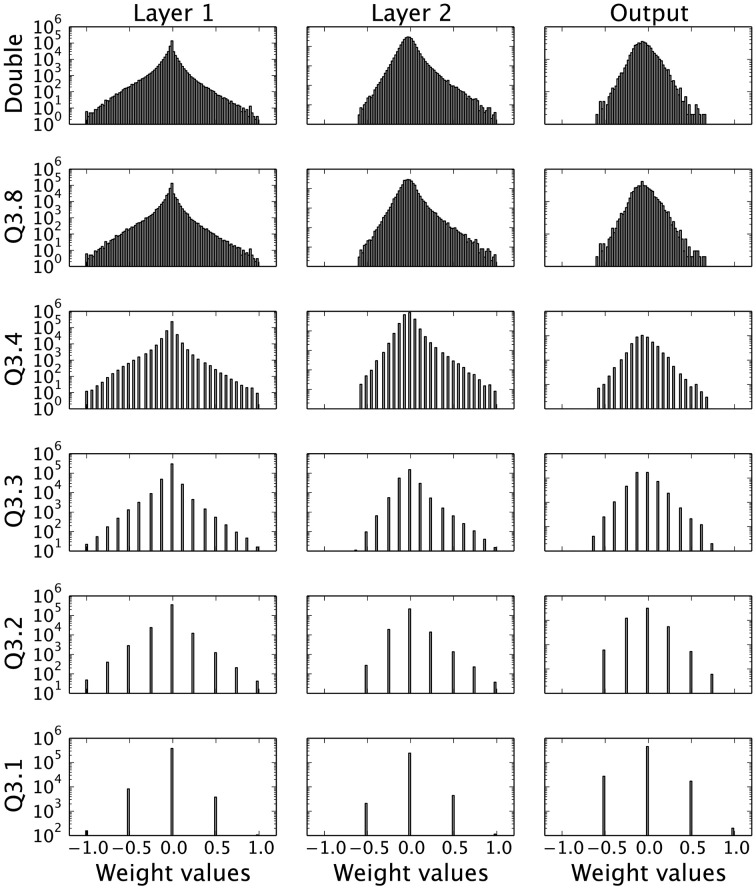
**Weight distributions for different bit precision levels and DBN layers**. Each row represents different fixed-point weight precisions, while each column represents a layer of the DBN, starting from Layer 1 (left), Layer 2 (middle) to the Output Layer (right). Despite the different discretization levels, the overall shape of the weight distribution is conserved.

There are a few tools that can be employed to investigate how the distribution of the reduced bit precision weights nonetheless manages to maintain a substantial amount of the network's classification performance. Firstly, the initial question is to investigate whether this reduction in bit precision qualitatively maintains the same weight distribution as the original. Figure 5 shows that the quasi-continuous distribution of weights obtained for double-precision becomes increasingly discretized as the precision *f* decreases. In the extreme case of a Q3.1 representation, the weight values are quantized to ± 0.5, ± 1, and 0, but nonetheless seem to reflect the shape of the original distribution.

**Figure 6:**
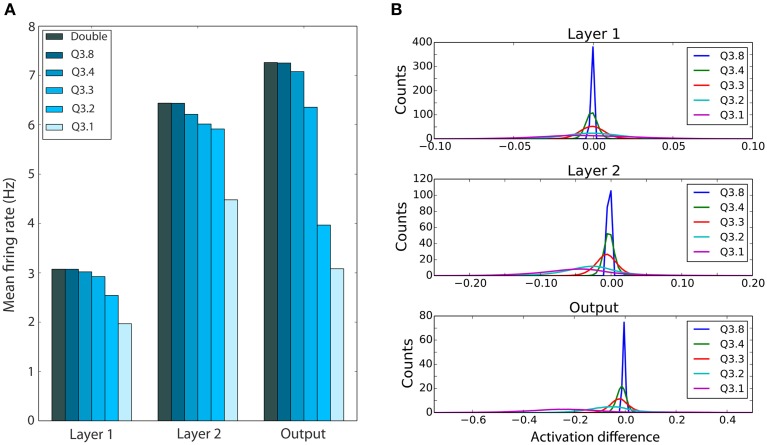
**Effect of reduced bit precision on firing rates in the DBN and neuron activations**. **(A)** Mean firing rate of each layer of the network for weights with different bit precisions, using an input rate of 1500 Hz. Lower precision levels, which lead to more weights at zero, cause lower firing rates within the network. **(B)** Distribution of the mean difference between the activation of a neuron with double precision weights and neurons using weights with different bit precision levels. Shown are distributions over all test samples. The difference, although peaked near zero, increases for higher layers, and shows a trend toward reduced activations.

However, even with these similar shaped weight distributions, neurons' output firing rates may become dramatically altered by the subtle coercion of weights to become more similar to each other. For this, refer to Figure 6A which shows that for even high levels of quantization, the mean output spike rate per neuron for each of the three layers remains quite constant down to Q3.3, before a clear drop in the mean firing rate is observed. This trend is seen for all three layers, but is stronger in higher layers.

Finally, since these firing rates are approximately the same, we investigated whether the net activations of the neurons for the same inputs remain similar despite the quantized weight structure. Since the net activations are sums over large numbers of synapses, any rounding effects could just average out, which would help to explain the maintenance of performance with lower precision weights observed in Figure 4. To investigate this proposal, Figure 6B plots the distribution of mean differences in the net activation between neurons with double precision weights and lower precision weights for neurons in different layers. Note how in all layers the net difference in activation is much smaller than the full range of firing rates (−1 to 1), and though the width of the distribution increases as the accuracy drops, most of the weight of this histogram is concentrated around zero difference in activation. This does imply that most neurons end up with approximately the same input activation under the quantized weights, and suggest that indeed the rounding differences tend to cancel out their effects.

### 3.3. Robustness to variance of synaptic weights

If spiking DBNs are to be implemented in analog circuits, they have to be robust to mismatch due to the fabrication process of transistors. This process causes random variations of physical quantities (for e.g., currents) of equally sized devices and comes from sources such as the random variations in the threshold, width and length of the transistor during fabrication (Pelgrom et al., 1989; Pavasovic et al., 1994; Kinget, 2005). Measurements of these random variations is a standard practice for all silicon process technologies and is indicated by the measured standard deviation assuming a Gaussian distribution of transistor currents. Mismatch can become a factor that makes the performance of a hardware network very different from a digital simulation, and needs to be taken into account when designing mixed-mode neuron and synaptic circuits in analog/digital multi-neuron chips (Serrano-Gotarredona et al., 2008; Brink et al., 2013; Wang and Liu, 2013; Moradi and Indiveri, 2014). The influence of fabrication variance is also a concern for circuits that use memristive technology (Alibart et al., 2012), where the coefficient of variation (CV) of the devices can exceed 40% in resistive values for academic technologies. The dependence of the mismatch transistor current variance on the transistor area and the current magnitude has been quantified in a CMOS 0.35 μm process (Serrano-Gotarredona and Linares-Barranco, 1999). In order to implement the maximal number of neurons and synapses possible per chip area means that a circuit with as few transistors as possible is needed to implement their functionalities, and transistors should be small-sized. Unfortunately, the latter can lead to very large CV (>100%).

In order to understand the effect of parameter variance in analog circuits on the performance of spiking DBNs, simulations were performed where synaptic weights were randomly perturbed according to the mismatch model for a particular analog synaptic circuit. In this analysis, we chose the digital-to-analog converter (DAC) synapse used on various neural chip implementations (Wang and Liu, 2006; Vogelstein et al., 2007; Schemmel et al., 2010; Linares-Barranco et al., 2011; Wang and Liu, 2011; Moradi and Indiveri, 2014). The number of bits in the DAC synapse is equivalent to the *f* value in the Q*m*.*f* format used for the bit precision. In this case, the quantized weight levels available are *I*_*ref*_/2^−*f*^ where *I*_*ref*_ is the maximum current that is equivalent to the maximum synaptic weight.

Mismatch measurements from 50 copies of a particular five-bit current DAC circuit in Linares-Barranco et al. (2011) show a standard deviation around 7.77%. While the *I*_*ref*_ can be calibrated to minimize the effect of the mismatch, we assume that there is no calibration because it would be too expensive to calibrate the many weights of a DBN network. In the case where a single DAC is used for positive and negative weights, then one bit is used as the sign bit.

**Figure 7:**
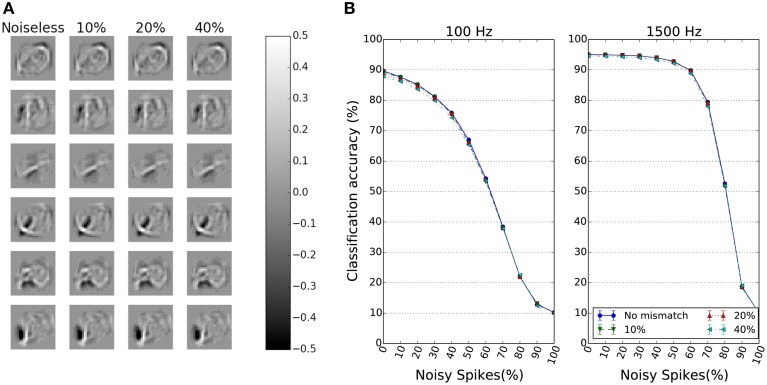
**Effect of Gaussian weight variance on the performance of spiking DBNs**. **(A)** Receptive fields of 6 representative neurons in the first hidden layer after perturbation with Gaussian weight variance of different CVs. **(B)** Impact of Gaussian weight variance on classification accuracy. The performance is plotted as a function of input noise levels for two different input rates and different weight distribution CVs. Despite the high weight variance, the performance stays high and remains robust to input noise. All weights are set by 5 bit DAC synapses (one bit is the sign bit). Results over 4 trials.

We ran simulations on a network where each synapse has a five-bit DAC. The maximum current *I*_*ref*_ = 1 nA and one bit is used as the sign bit. The circuit noise sources such as flicker noise and thermal noise are ignored in these simulations both because of the extensive time for such simulations and the dependence on the actual device sizes of the synapse. The mismatch of the transistor that supplies the maximum current for the DAC of a synapse is assumed to have a CV of 10 or 40%. The effect of applying a CV of 40% to the weights of the receptive fields of six representative neurons in the first layer of the DBN is shown in Figure 7A. Despite this high CV, the receptive fields look very similar.

The robustness of a network with this DAC precision to Gaussian variance on *I*_*ref*_ is illustrated in Figure 7B. The plots show again the performance as a function of input noise, and for two different input rates. The effect of increased Gaussian weight variance is minimal as can be seen from the different curves. As the CV increases to 40%, the classification accuracy in both the cases of 100 and 1500 Hz input rates, decreases by < 1% from the noiseless Q3.4 bit precision case.

### 3.4. Comparison of hardware performance to simulations

In order to validate the simulation results presented in Section 3.1 on actual hardware platforms, we ran simulations of spiking DBNs on the fixed-point, event-based digital platform SpiNNaker. For the DBN with two hidden layers, Figure 1B, the double floating-point weights were truncated to a Q3.8 fixed-point representation, and the input rates used to encode the MNIST images into spike trains were set to 1500 Hz. The classification accuracy achieved by the SpiNNaker platform on the MNIST testing set, is 94.94% when the input noise is set to 0 and 88.66% when the input noise is set to 60%. This is in good accordance with the noise-free simulation results obtained by O'Connor et al. (2013), and classification accuracies for simulations on Brian (Goodman and Brette, 2008), which reach 94.95 and 88.66%, respectively, for the same weight precision and input noise (Figure 4). This spiking DBN ran on a single SpiNNaker chip and generated an activity of <1 million synaptic events (SE) per second, which is well below the 36.8 million SE a SpiNNaker chip can process (Stromatias et al., 2013). The comparison of results is summarized in Table 2.

**Table 2:** **Classification accuracies for hardware and software simulations with limited bit precision, two different input noise levels, and input rates of 1500 Hz**.

**Input noise (%)**	**Brian (%)**	**SpiNNaker (%)**
0	94.955	94.94
60	88.665	88.66

*The first column shows results for a software simulation in Brian with Q3.8 precision, the second column shows results for SpiNNaker, which uses the same fixed point representation of weights. Differences in performance are almost negligible*.

Our results indicate that the difference in the classification accuracy between SpiNNaker and Brian for the same bit resolution, input rates, and input noise levels is almost negligible and in the order of 0.01%. Moreover, the difference between the software simulation that utilizes double floating-point weights and SpiNNaker with Q3.8 fixed-point weights is 0.06%, which is in agreement with a previous study (Stromatias et al., 2015b).

### 3.5. Training of spiking DBN using different bit precision

Beyond the method of rounding the weights of a DBN after training has been completed, this work introduces two additional approaches to create the lower-precision weights, and optimize the performance for low-precision simulations. Intuitively, the motivation for these novel methods arises from the idea that networks that incorporate knowledge about the eventual low-precision representation during training may be able to perform better under those low-precision conditions than networks that have been optimized under the assumption of higher precision.

The first proposed method, called *iterative rounding*, is similar to the fixed-point method mentioned in Courbariaux et al. (2015), in which the result of a computation is rounded whenever it is stored. This method, however, refers to the case when the forward pass of computing activities of neurons in all layers, and the computation of gradients for learning are performed with full precision, and only the weight is kept in reduced precision. For iterative rounding, the full-precision weight update is calculated from the contrastive divergence algorithm (see Section 2.2), and applied directly to the low-precision weights. After the full-precision weight update has been applied, the value is then rounded to the closest low-precision representation of that weight and stored.

However, one challenge with this approach is that the gradient update may be too small to change the values of low-precision weights. To address this potential difficulty, this paper introduces a key, novel method called *dual-copy rounding*, which stores both a low-precision and a high-precision representation of weights. This method incorporates knowledge about the eventual low-precision representation of the tested network as well as supporting the small, but highly important accumulation of error gradients over multiple iterations. For this method, two copies of the weight matrix *W* are maintained during training: a high-precision weight matrix (*W*_*H*_) and a low-precision weight matrix (*W*_*L*_), which is stored in Q*m.f* numerical format. Learning proceeds as in (O'Connor et al., 2013), but the activities of the hidden layer and the visible layer after sampling are obtained using the low-precision weights *W*_*L*_. The contrastive divergence update for *W*_*H*_ in Equation (4) is thus parameterized as Δ*w*(*W*_*L*_), and after the update both weight matrices are processed as

(6) $$W_{H}=\{\begin{array}{ll} -2^{m} & \text{where }W_{H}\leq -2^{m}\\ W_{H} & \text{where }-2^{m}<W_{H}<2^{m}\\ 2^{m} & \text{where }W_{H}\geq 2^{m} \end{array}$$

(7) $$W_{L}=\text{round}(2^{f}\;\cdot \;W_{H})\;\cdot \;2^{-f}$$

where 2^*m*^ represents the largest possible value that can be stored in the Q*m.f* format. Importantly, note that the low-precision weight matrix *W*_*L*_ is used to sample from the network, while the weight update is applied to the higher-precision representation *W*_*H*_, and *W*_*L*_ is obtained via rounding. As in standard contrastive divergence, the weight update is calculated from the difference of pairwise correlations of the data-driven layers and the model-driven sample layers. Here, although the activations are calculated from the low-precision weights, the updates are accumulated in the high-precision weights. Then, the weights are checked to be within the maximum bounds of the given resolution (Equation 6) for the given fixed-point precision. Finally, the weights are copied over into the low-precision matrix (Equation 7). The learning can then proceed for another iteration, using the new updated low-precision weight matrix *W*_*L*_. The additional cost of dual-copy rounding is to store a second weight matrix in memory, which is typically not a limiting factor for off-chip learning.

**Figure 8:**
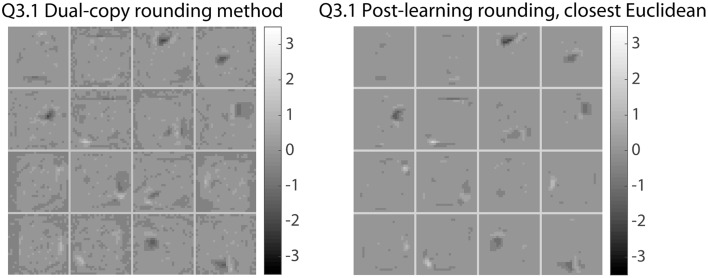
**Impact of different rounding methods during learning on learned weight representations**. Comparison of first-layer weights in networks trained with the dual-copy rounding method (left) and the post-learning rounding method (right). The weights shown here are representative samples from 16 clusters of weight vectors in the learned dual-copy rounding weight matrix. On the right, the weights from the post-learning rounding weight matrix that are most similar to these chosen weights are displayed. The dual-copy rounding method is able to preserve much more fine structure, compared to simply rounding the network weights after training, and is thus more suitable for training networks that will be executed with lower bit precision weights.

For qualitative differences, observe the weights shown in Figure 8. In order to show representative samples, the learned weights in the first layer from the dual-copy rounding method were clustered into 16 categories, and the post-learning rounding method weights with the closest Euclidean distance to these cluster exemplars were identified and plotted on the right. The dual-copy rounding method preserves significantly more fine-grained structure, which would be lost with other rounding methods.

For a quantitative analysis of the differences in performance, the classification accuracy in the MNIST task using different bit precisions and different rounding methods was measured. For performance reasons, the classification performance for this section unlike the other sections in this paper occurred in rate-based (non-spiking) conditions, but using the same training procedure for spiking neurons.

**Figure 9:**
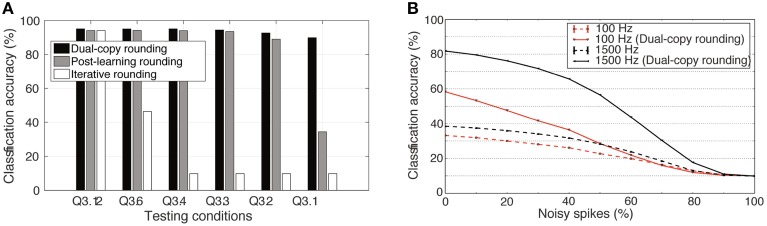
**Impact of different rounding methods during training on network performance with reduced weight bit precision**. **(A)** Effectiveness of the dual-copy rounding weight training paradigm. Training at full precision and later rounding performs consistently worse than the dual-copy rounding method introduced in this paper. Rounding the weights during training can prevent learning entirely at low-precision regimes. The results show averages of five independent runs with different random seeds. **(B)** Increase in classification accuracy of a spiking DBN with Q3.1 precision weights due to the dual-copy rounding method for input rates of 100 and 1500 Hz. Results over four trials.

As there was no performance loss in the Q3.12 representation compared to full double-precision, this was taken as the full-precision reference point. Figure 9 shows the effect of the three investigated training methods on the classification accuracy, when testing the weight matrix at different levels of bit precision. Rounding a high-precision weight matrix does work effectively, but can fail for lower-precision weights. Unfortunately, the iterative rounding method of training works extremely poorly for low-precision cases; the weight update size is simply less than the precision of the weight, so learning halts entirely after the error gradient falls below a certain threshold.

Impressively, however, Figure 9A shows the clear advantage of the dual-copy training. Across all precision levels, incorporating information about the lower-precision weight matrix into the training yields noticeable and consistent improvements. This improvement increases as precision decreases, so that at the Q3.1 representation level, where there is only a single sign bit and three bits of representation per weight, the network is able to achieve an impressive 91.35% accuracy in the highest-performing case. Under spiking conditions, the performance of this network drops to 82%, Figure 9B. While this is substantially lower than the rate-based performance, it is still twice the accuracy of the default post-learning rounding method (see Figure 4), and future work will determine improved ways to maintain the rate-based performance under spiking conditions.

## 4. Discussion

After outperforming other machine learning approaches on typical benchmark tasks in vision and audition, transferring Deep Learning techniques into marketable applications has become the next big target, and is supported by large ongoing industrial efforts. One of the biggest challenges is making the classification results of deep networks available in real time, which is necessary to improve user experience for relevant applications such as speech recognition or visual object recognition. It has become clear that apart from cloud computing solutions, which require additional communication overheads, the development of special purpose hardware to support deep networks is one of the most promising routes, in particular for mobile platforms with limited resources. Spiking deep networks have demonstrated very favorable properties in terms of latency and scaling (O'Connor et al., 2013; Stromatias et al., 2015a,b), and are a good match for ongoing efforts to advance the design of both digital and mixed-signal neuro-inspired hardware platforms (Pham et al., 2011; Pfeil et al., 2013; Furber et al., 2014; Merolla et al., 2014b; Liu et al., 2015). Such implementations range from custom mixed-signal multi-neuron platforms to more general digital platforms such as FPGAs and SpiNNaker. Thus, the present investigation of the impact of digital bit precision, input rates, and analog transistor mismatch on the performance of the hardware implementation of a spike-based DBN is of high relevance to justify the development of larger neuromorphic platforms that support larger networks. This is particularly relevant since theory tells us that the performance of DBNs increases with the numbers of layers (Hinton and Salakhutdinov, 2006), although this does not necessarily generalize to multi-layered networks with reduced weight precision. Note that here we are focusing on mapping networks that have been trained off-chip to neuromorphic hardware, rather than training networks on chip. This is because current training methods for deep networks from large datasets are optimized for exploiting conventional computing technology such as GPUs, but the execution on event-based platforms yields efficiency advantages as discussed previously.

Our results show indeed that spike-based DBNs exhibit the desired robustness to input noise and numerical precision. The classification performance of the spike-based DBN on the MNIST digit database holds up even for bit precisions down to Q3.3, which requires significantly fewer bits to represent the large parameter space of DBNs than in typical CPU systems. For example, the two-layer DBN has 642,510 synapses, which would require 4.9 MBytes if they were stored in double floating-point precision (64 bits per weight). This reduces to only 0.46 MByte, or <10% if weights are stored in Q3.3, i.e., six bit per weight precision. Even more, one of the effects of the reduced precision is that many of the weights, which typically are distributed around zero, actually become zero. For low precisions, this means that the performance can be maintained, although more than 50% of the weights become zero. Thus, these synapses are ineffective, and more than half of the weights can be ignored. This not only saves time during execution, because of the savings in the memory lookup time for the synaptic weights in the case of a digital platform implementation, but also means that larger networks can be realized on the same hardware, because only a smaller percentage of the weights actually need to be represented. A validation was achieved by running the DBN for MNIST classification on the biologically-inspired massively-parallel fixed-point SpiNNaker platform, which uses less precise weights than standard software implementations. We have shown that the resulting performance of the network implemented on SpiNNaker is very close to the results from the software simulation with only a 0.06% difference, despite the fact the SpiNNaker uses fixed-point arithmetic.

For implementations on custom mixed-signal hardware systems one has to deal with the constraint that they can only offer reduced numerical precision in the synaptic weights (Neftci et al., 2011; Liu et al., 2015). The level of mismatch in the individual synapses can be taken into account during design and reduced by methods such as clever layout strategies and increasing the transistor area. Reduction of mismatch through increasing transistor area is effective (Kinget, 2005) but it increases the overall area of the synapse. Mismatch calibration methods through for example, a global Digital-to-Analog Converter block (Oster et al., 2008) can be introduced to combat this mismatch after fabrication but the calibration itself can take a long time. The mismatch influence is also greater in low-power dissipation systems, where the transistors are usually operated in the subthreshold domain for reduced transistor current (Kinget, 2005; Linares-Barranco et al., 2011). Our results show that up to 40% of the CV for a normal distribution of mismatch can be tolerated for the network to produce approximately the same level of performance. Thus, the effects of hardware-induced imperfections seem to rather cancel out than accumulate in spiking DBNs. This study adds to current on-going studies into computational spiking network models that are robust to some level of device mismatch including that of networks with memristive devices and smaller-scale multi-neuron networks with additional spatio-temporal dynamics (Liu and Douglas, 2004; Arthur and Boahen, 2007; Vogelstein et al., 2007; Pfeil et al., 2012; Basu et al., 2013; Brink et al., 2013; Querlioz et al., 2013; Wang and Liu, 2013; Moradi and Indiveri, 2014).

All our results show that the networks tolerate high levels of noise in the input spike trains. In almost all cases, and for different bit precisions, input noise of up to 60% can be tolerated before the performance deteriorates. The performance also shows some tolerance to the input spike rate. This is important because we intend that the hardware implementations will be interfaced to spike-based hardware visual and auditory sensors (Liu and Delbruck, 2010; Brändli et al., 2014; Liu et al., 2014; Posch et al., 2014). What can be observed is a tradeoff between higher spike rates, which require more computation but lead to better CA and lower latency; and lower spike rates which yield a more energy-efficient system. With higher spike rates, the network is a closer approximation to the analog DBN, and thus its performance is better matched. The inherent tradeoff between input spike rates, latency, and classification accuracy has also been validated on the SpiNNaker platform (Stromatias et al., 2015b).

Although spiking DBNs are remarkably tolerant to porting their parameters to low-precision platforms, we have shown that their performance can even be improved if the constraints of the hardware are taken into account already during training. The novel *dual-copy offline learning* rule introduced in the present article increases the performance of the network for lower bit precisions. In the case of very low Q3.1 precision, the network performance was shown to double compared to other common methods (post-learning rounding and iterative rounding), thus allowing a very fast and cheap hardware implementation of a DBN with reasonable performance levels. Because the dual-copy rounding method focuses on acceptable performance using very efficient and low-precision networks, it operates in regimes such as Q3.1 where methods such as iterative rounding entirely fail to train the network effectively. The method requires storage of a second weight matrix, and uses the two sets of weights in two different contexts—computing activations and learning. This is typically not a problem in the off-chip learning scenario that is the focus of this paper, but might be challenging for on-chip learning, in particular on hardware where synapses are physically emulated in silicon. Once trained, the single bit precision analog network can potentially be implemented on a digital neuron platform such as TrueNorth (Merolla et al., 2014b), using the Gibbs sampling methods recently described in Das et al. (2015).

Future work will investigate the scaling behavior of deeper DBN architectures and other types of deep architectures on custom analog/digital multi-neuron systems or digital platforms with limited precision. Since training remains the most computationally expensive task with DBNs, it will be interesting to study how event-based learning rules on neuromorphic platforms can contribute to speeding up this process. Online learning rules such as the recently proposed event-based Contrastive Divergence learning rule (Neftci et al., 2014) for training a spike-based DBN can in principle utilize neuromorphic platforms for DBN training, and will have to deal with similar hardware constraints as addressed in the present article. Current neuromorphic plasticity implementations are often limited to various forms of STDP, but more general plasticity frameworks such as the one recently proposed in Galluppi et al. (2014) would provide the necessary flexibility to also test variations of contrastive divergence or related learning rules for DBNs on massively parallel brain-inspired hardware platforms.

## Author contributions

ES performed the software network simulations, data analysis, and the implementation on SpiNNaker. DN also performed the software network simulations, data analysis, and the experiments with the training networks. MP contributed to the design of the experiments, analysis and presentation of the work. FG contributed to the infrastructure on the SpiNNaker system for enabling the network implementation on SpiNNaker. SF contributed to the infrastructure of the SpiNNaker system. SL contributed to the conception and design of the experiments, data analysis, and presentation of the work.

### Conflict of interest statement

The authors declare that the research was conducted in the absence of any commercial or financial relationships that could be construed as a potential conflict of interest.
